# Development of a Monoclonal Antibody-Based icELISA for the Detection of Ustiloxin B in Rice False Smut Balls and Rice Grains

**DOI:** 10.3390/toxins7093481

**Published:** 2015-08-28

**Authors:** Xiaoxiang Fu, Ali Wang, Xiaohan Wang, Fengke Lin, Lishan He, Daowan Lai, Yang Liu, Qing X. Li, Ligang Zhou, Baoming Wang

**Affiliations:** 1 College of Agronomy and Biotechnology, China Agricultural University, Beijing 100193, China; E-Mails: xiaoxiaofu@cau.edu.cn (X.F.); wangali526@163.com (A.W.); wangxiaohan99@126.com (X.W.); fengkelin.123321aa@foxmail.com (F.L.); hls0305@136.com (L.H.); dwlai@cau.edu.cn (D.L.); 2 Institute of Agro-Products Processing Science and Technology, Chinese Academy of Agricultural Sciences, Beijing 100193, China; E-Mail: liuyang01@caas.cn; 3 Department of Molecular Biosciences and Bioengineering, University of Hawaii at Manoa, Honolulu, HI 96822, USA; E-Mail: qingl@hawaii.edu

**Keywords:** ustiloxin B, ELISA, mycotoxin, phytotoxin, rice false smut, *Villosiclava virens*

## Abstract

Rice false smut is an emerging and economically-important rice disease caused by infection by the fungal pathogen *Villosiclava virens*. Ustiloxin B is an antimitotic cyclopeptide mycotoxin isolated from the rice false smut balls that formed in the pathogen-infected rice spikelets. A monoclonal antibody (mAb) designated as mAb 1B5A10 was generated with ustiloxin B—ovalbumin conjugate. A highly-sensitive and specific indirect competitive enzyme-linked immunosorbent assay (icELISA) was then developed. The median inhibitory concentration (IC_50_) of the icELISA was 18.0 ng/mL for the detection of ustiloxin B; the limit of detection was 0.6 ng/mL, and the calibration range was from 2.5 to 107.4 ng/mL. The LOD/LOQ values of the developed ELISA used for the determination of ustiloxin B in rice false smut balls and rice grains were 12/50 μg/g and 30/125 ng/g, respectively. The mAb 1B5A10 cross-reacted with ustiloxin A at 13.9% relative to ustiloxin B. Average recoveries of ustiloxin B ranged from 91.3% to 105.1% for rice false smut balls at spiking levels of 0.2 to 3.2 mg/g and from 92.6% to 103.5% for rice grains at spiking levels of 100 to 5000 ng/g. Comparison of ustiloxin B content in rice false smut balls and rice grains detected by both icELISA and high performance liquid chromatography (HPLC) demonstrated that the developed icELISA can be employed as an effective and accurate method for the detection of ustiloxin B in rice false smut balls, as well as rice food and feed samples.

## 1. Introduction

**Figure 1:**
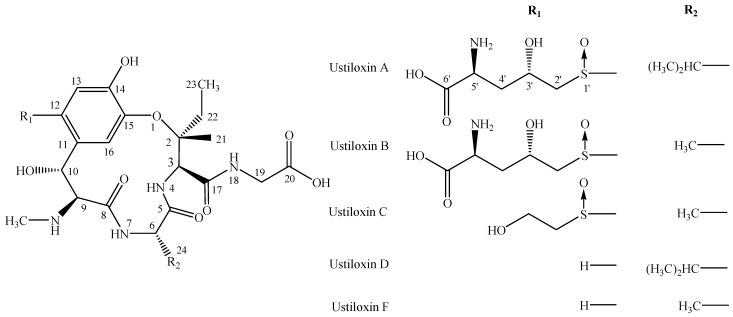
Chemical structures of ustiloxins.

Rice false smut, caused by the ascomycete fungus *Villosiclava virens* (Nakata) Tanaka & Tanaka (anamorph: *Ustilaginoidea virens* Takahashi) [1], is one of the most destructive rice (*Oryza sativa* L.) fungal disease in many rice-growing areas worldwide over the past few years [2,3,4,5]. This fungus infects the rice filament and transforms individual grains of the panicle into greenish balls (namely rice false smut balls) covered by powdery dark-green chlamydospores [4,6]. The rice false smut was previously regarded as a minor disease, but in recent years, the disease has become significant due to the heavy application of nitrogenous fertilizers and widespread cultivation of hybrid cultivars without high-level resistance sources in the existing rice germplasm [7,8], resulting in yield loss, rice grains, feed contamination and, even more important, generating mycotoxin poisoning of humans and animals [9,10,11]. Both ustiloxin A and the crude fraction obtained from the water extract of rice false smut balls caused liver and kidney damage in mice [12]. The cytotoxic activity of the ustiloxins has been approved to be antimitotic by inhibition of the microtubule assembly and cell skeleton formation [13]. Two kinds of mycotoxins, namely ustiloxins and ustilaginoidins, have been isolated and identified from rice false smut balls and false smut pathogen [10,14,15]. The ustiloxin family, consisting of ustiloxins A, B, C, D and F (Figure 1), belongs to the cyclopeptides containing a 13-membered cyclic core structure with a phenol ether linkage, and ustiloxin A is the most toxic and predominant among them, followed by ustiloxin B [9,16,17,18]. It has been reported that ustiloxins had antimitotic activity by inhibiting microtubule assembly and cell skeleton formation of plant and animal cells [13,19,20]. The crude water extract of rice false smut balls was found to cause necrosis of the liver and kidney in mice quite similar to that observed in lupinosis caused by phomopsin A, a mycotoxin produced by *Phomopsis leptostromiformis* [12,21]. Meanwhile, ustiloxins functioned as the phytotoxins by inhibiting the radicle and plumule growth during seed germination of rice, wheat and maize, inducing an abnormal swelling of the seeding roots and resulting in the growth reduction, necrotic and dead frond tissue to duckweed (*Lemna pausicostata*) [5,9,16]. The content of ustiloxin A in rice grains from rice false smut disease regions ranged from 0.0073 to 4.33 µg/g [22]; while ustiloxins A and B content was much higher in rice false smut balls, ranging from 0.13 to 1.08 mg/g [18,23]. Therefore, both rice false smut balls and false smut pathogen-contaminated rice food and forage have created concerns for food and feed safety [10].

The evidence from hazard assessment to evaluate the risk of ustiloxins in food and feed is still inadequate. One of the most important aspects for the hazard assessment of ustiloxins is to develop a rapid, sensitive, accurate and simple analytical method. To analyze ustiloxins, previous studies have developed some methods, including high-performance liquid chromatography (HPLC) for ustiloxins A and B [18,24], liquid chromatography-mass spectrometry (LC-MS) for ustiloxins A, B and D [18,22] and indirect competitive enzyme-linked immunosorbent assay (icELISA) for ustiloxin A [23]. Conventional instrumental methods are used for an accurate and reliable quantification of analytes. Despite the considerable advantages of chromatographic techniques, all of these methods require expensive equipment and highly skilled operators and are unsuitable for routine screening analyses [25,26]. Thus, it is necessary to establish a simple, rapid, low-cost and sensitive method for routine screening for monitoring purposes. Immunochemical determinations, such as enzyme-linked immunosorbent assays (ELISAs), applying antigen-antibody interaction showing a specific response against a particular substance, bring the detection of mycotoxins many advantages. Concretely, the analytical methods are capable of rapid and simple detection of a trace of mycotoxins in a sample composed of complex matrices, and furthermore, they can simultaneously handle many samples and do not require expensive instruments. These features convert ELISAs into very powerful tools for mycotoxin analysis [27,28,29].

To our knowledge, there was no report about ELISA for ustiloxin B analysis except our previous report for ustiloxin A determination [23]. In the present study, we developed a rapid, sensitive and specific icELISA based on a monoclonal antibody against ustiloxin B, and it was evaluated for the analysis of ustiloxin B in the rice samples, including rice false smut balls and rice grains. The results of ustiloxin B content in the samples determined by both icELISA and HPLC methods were also compared.

## 2. Results and Discussion

### 2.1. Identification of Hapten-Protein Conjugates

The hapten load on carrier proteins was estimated via UV-VIS spectral measurement, which could reflect the structural characteristics of molecules containing π-electrons or non-bonding electrons [30]. The results showed that the haptens were successfully coupled to the carrier proteins. The molar ratios of ustiloxin B to proteins were estimated to be 7:1 and 6:1 for ustiloxin B-bovine serum albumin (UB-BSA) and ustiloxin B-ovalbumin (UB-OVA), respectively.

### 2.2. Production and Characterization of mAbs

To determine the titer (the antiserum dilution that gave an absorbance of 1.0 in the noncompetitive assay conditions) and inhibition of antisera against ustiloxin B, the blood samples were obtained from the mice immunized with the immunogen (UB-OVA) on Day 3 after the fifth immunization. The prepared antisera showed high affinity to ustiloxin B. When the concentration of the coating antigen (UB-BSA) was 0.25 µg/mL and the antisera were diluted with 0.01 M pH 7.5 phosphate buffer containing 0.9% NaCl (*w/w*), 0.1% Tween-20 (*v/v*) and 0.5% gelatin (*w/v*) (PBSTG) at 1:2000, the best inhibition by ustiloxin B standard solution at 2000 ng/mL was 80% for antisera from seven mice, and the titer of antisera was higher than 8000. The mouse that produced antisera that had the best inhibition and a high enough titer had its spleen cells collected for *in vitro* hybridoma cell production. The hybridoma cell lines screened by icELISA that showed high affinity and good inhibition were cloned using limiting dilution. One clone, named 1B5A10, with the best inhibition by ustiloxin B, was expanded for ascites production. The titer of the ascites was 1.28 × 10^5^. The monoclonal antibody (mAb) from 1B5A10 was confirmed as an immunoglobulin G1 (IgG1) isotype.

### 2.3. Development of icELISA

#### 2.3.1. Optimization of icELISA Conditions

To optimize the conventional icELISA, various dilutions of the coating antigen UB-BSA (0.06 to 2.00 µg/mL) and mAb (0.13 to 2.00 µg/mL) from the clone 1B5A10 were screened by checkerboard titration. The optimum concentrations of the coating antigen, purified mAb and anti-mouse immunoglobulin G conjugated with horseradish peroxidase (IgG-HRP) for icELISA were at 0.5, 0.5 and 1.0 μg/mL, respectively. An icELISA under the optimized conditions was then developed.

#### 2.3.2. Assay Sensitivity

**Figure 2:**
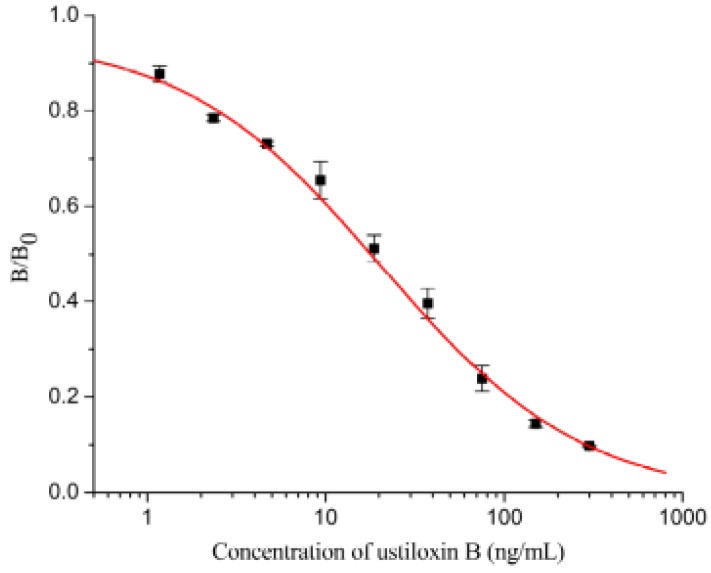
Inhibition curve of ustiloxin B in indirect competitive ELISA (icELISA) format based on mAb IB5A10 (each value represents the mean of triplicate ± standard deviations; B_0_ and B are the absorbance values at 492 nm in the absence and presence of ustiloxin B, respectively).

The icELISA measurements were conducted with a series of concentrations (0, 1.17, 2.34, 4.69, 9.38, 18.75, 37.5, 75, 150, 300 ng/mL) of ustiloxin B dissolved in PBSTG under the optimal conditions. A representative inhibition curve (Figure 2) for ustiloxin B generated by icELISA based on mAb IB5A10 was established. The median inhibitory concentration (IC_50_) of the icELISA was 18.0 ng/mL. The limit of detection was 0.6 ng/mL (10% inhibition). The calibration range, based on 20% to 80% of inhibition of the binding of mAb 1B5A10 to the immobilized hapten-BSA, was from 2.5 to 107.4 ng/mL.

#### 2.3.3. Antibody Specificity

**Figure A1:**
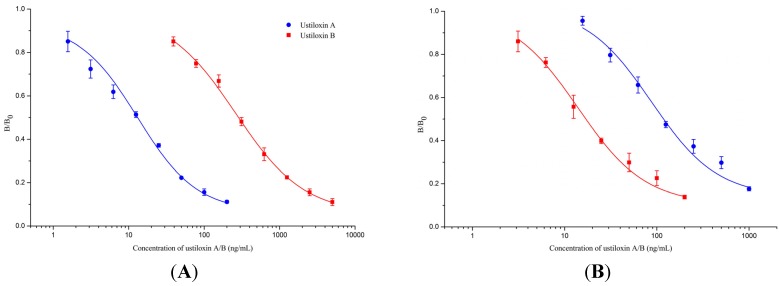
Inhibition curves of ustiloxin A/B in icELISA format (each value represents the mean of triplicate ± standard deviations. B_0_ and B are the absorbance values at 492 nm in the absence and presence of ustiloxin A/B, respectively). (**A**) Inhibition curve of ustiloxin A/B in icELISA format based on 2D3G5. The cross-reactivity with ustiloxin B was 4.1% for mAb 2D3G5, as previously reported [23]. (**B**) Inhibition curve of ustiloxin A/B in icELISA format based on mAb 1B5A10. The cross-reactivity with ustiloxin A was 13.9% for mAb IB5A10.

Both ustiloxins A and B are the predominant ustiloxins in rice false smut balls and rice grains [9,18]. As ustiloxins A and B are available at present, the specificity of mAb 1B5A10 against ustiloxins A and B was evaluated. The structure of ustiloxin B is the most similar to ustiloxin A among the five known ustiloxins. There is a minor difference with two methyl groups at the C-24 position between ustiloxins A and B (Figure 1). In the preparation of hapten-protein conjugates, ustiloxin B was conjugated with carrier proteins via –NH_2_ at the C-5ʹ position with the glutaraldehyde method. In general, there is some correlation between the position conjugated to the carrier protein and the recognition of epitopes on the hapten by the prepared antibodies. The epitopes distant from the site of conjugation tend to be well recognized by antibodies, whereas epitopes neighboring the coupling site tend to be less well recognized. Although a structural difference between ustiloxins A (HR-ESI-MS, *m*/*z* 674.26859 [M + H]^+^) and B (HR-ESI-MS, *m*/*z* 646.23751 [M + H]^+^) exists on the opposite side of the conjugation site, the high molecular weight of the cyclopeptide ustiloxins might affect the specificity of mAb 1B5A10, resulting in worse recognition [31,32]. The IC_50_ values of ustiloxins A and B were 122.6 and 17.1 ng/mL, respectively. There was still 13.9% cross-reactivity with ustiloxin A relative to ustiloxin B (Figure A1). Ustiloxins C, D and F are structurally very different from ustiloxins A and B (Figure 1) and are less abundant than ustiloxins A and B. Therefore, ustiloxins C, D and F presumably have minor cross-reactivities and may not interfere with the developed icELISA, which, however, needs to be verified.

#### 2.3.4. Recoveries of Ustiloxin B from the Spiked Samples

To test the reliability and accuracy of the assay, the quantity of ustiloxin B spiked in rice false smut ball samples (Hunan, China) and rice grain samples (Beijing, China) was determined by icELISA and HPLC. The LOD/LOQ values of the developed ELISA used for the determination of ustiloxin B in rice false smut balls and rice grains were 12/50 μg/g and 30/125 ng/g, respectively. The average recoveries of ustiloxin B from the spiked rice false smut balls ranged from 91.3% to 105.1% (detected by icELISA) and 95.2% to 109.2% (detected by HPLC) (Table 1). A good correlation between the results of ustiloxin B content detected by HPLC (*Y*) and icELISA (*X*) was observed with the linear regression equation of *Y* = 1.1425*X* − 0.0883 (*R*^2^ = 0.9987). For the blank rice grain sample, the content of ustiloxin B determined by ELISA was less than the LOD (*i.e*., 30 ng/g), showing no matrix interference for the developed assay. The average recoveries of ustiloxin B from the spiked rice grain samples determined by icELISA ranged from 92.6% to 103.5% (Table 2). As the content of ustiloxin B was too low in the spiked rice grain samples, it was undetectable by HPLC. The results demonstrated that the developed icELISA can be employed as an effective and accurate method for ustiloxin B analysis in rice false smut balls, as well as rice food and feed samples.

**Table 1:** Mean recoveries of ustiloxin B spiked to rice false smut ball samples detected by icELISA and HPLC.

Spiked Content of Ustiloxin B (mg/g)	icELISA		HPLC	
	Detected Content of Ustiloxin B (mg/g)	Mean Recovery of Ustiloxin B (%)	Detected Content of Ustiloxin B (mg/g)	Mean Recovery of Ustiloxin B (%)
0.0	0.37 ± 0.03	-	0.36 ± 0.02	-
0.2	0.55 ± 0.03	91.3 ± 10.2	0.55 ± 0.003	95.2 ± 9.5
0.4	0.76 ± 0.08	98.6 ± 13.3	0.75 ± 0.02	96.4 ± 7.9
0.8	1.21 ± 0.13	105.1 ± 21.6	1.23 ± 0.04	108.3 ± 6.3
1.6	1.86 ± 0.09	93.0 ± 2.8	2.11 ± 0.16	109.2 ± 10.8
3.2	3.46 ± 0.21	96.7 ± 7.0	3.85 ± 0.15	109.1 ± 4.8

Note: Recoveries were determined after subtraction of the background content of ustiloxin B. For icELISA analysis, the spiked concentrations of ustiloxin B in diluted (800 folds) solutions were 0, 5, 10, 20, 40 and 80 ng/mL. The detected concentrations of ustiloxin B in diluted (800 folds) solutions were 9.23, 13.66, 19.10, 30.26, 46.42 and 88.30 ng/mL. For HPLC analysis, the spiked concentrations of ustiloxin B in the extracted solutions were 0, 4, 8, 16, 32 and 64 µg/mL. The detected concentrations of ustiloxin B in the extracted solutions were 7.24, 10.94, 15.11, 24.57, 42.19 and 78.59 µg/mL. Data represent the means of triplicate ± standard deviations.

**Table 2:** Mean recoveries of ustiloxin B from spiked rice grain samples detected by icELISA.

Spiked Content of Ustiloxin B (ng/g)	Detected Content of Ustiloxin B (ng/g)	Mean Recovery of Ustiloxin B (%)
0	-	-
100	103 ± 3	103.5 ± 3.4
250	252 ± 8	100.8 ± 3.2
500	506 ± 24	101.2 ± 4.8
1000	926 ± 24	92.6 ± 2.4
2000	1984 ± 35	99.2 ± 1.8
4000	3947 ± 58	98.7 ± 1.5
5000	4906 ± 72	98.1 ± 1.4

Note: Recoveries were determined after subtraction of the background content of ustiloxin B. The spiked concentrations of ustiloxin B in diluted (50-fold) solutions were 0, 2, 5, 10, 20, 40, 80 and 100 ng/mL. The detected concentrations of ustiloxin B in diluted (50 folds) solutions were 2.07, 5.04, 10.12, 18.52, 39.68, 78.94 and 98.12 ng/mL. Data represent the means of triplicate ± standard deviations.

#### 2.3.5. Comparison of icELISA and HPLC for Analysis of Ustiloxin B in Rice False Smut Ball Samples and Rice Grain Samples

The content of ustiloxin B in the rice false smut ball samples collected from different areas of China was determined by icELISA and HPLC. The ustiloxin B content varied in rice false smut ball samples, ranging from 0.13 to 0.51 mg/g by HPLC and from 0.17 to 0.71 mg/g by icELISA (Table 3). The icELISA results were quite similar to, but slightly higher than those of HPLC. The correlation between the results of HPLC (*Y*) and icELISA (*X*) was obtained with the linear regression equation of *Y* = 0.6238*X* + 0.0573 (*R*^2^ = 0.9558). This correlation slope is approximately half of that (1.1425) of the ustiloxin B-fortified samples, which may be due to the cross-reactivity of the icELISA with the other ustiloxins, such as the main toxin ustiloxin A.

**Table 3:** Comparison of ustiloxin B content in rice false smut ball samples detected by icELISA and HPLC.

Place and Time of Rice False Smut Ball Sample Collection	Ustiloxin B Content (mg/g)	
	icELISA	HPLC
Chengdu, Sichuan, China; September 2014	0.17 ± 0.02	0.13 ± 0.02
Fengyang, Anhui, China; October 2014	0.33 ± 0.03	0.31 ± 0.01
Hefei, Anhui, China; October 2014	0.31 ± 0.04	0.28 ± 0.02
Hanshou, Hunan, China; October 2013	0.44 ± 0.04	0.33 ± 0.005
Linyi, Shandong, China; October 2013	0.71 ± 0.05	0.51 ± 0.01
Jianyang, Fujian, China; November 2012	0.39 ± 0.07	0.28 ± 0.03
Qionglai, Sichuan, China; September 2012	0.25 ± 0.03	0.20 ± 0.01
Changsha, Hunan, China; November 2011	0.64 ± 0.02	0.42 ± 0.02
Donggang, Liaoning, China; October 2010	0.20 ± 0.04	0.18 ± 0.02
Donggang, Liaoning, China; December 2011	0.27 ± 0.04	0.24 ± 0.01

Note: Data represent the means of triplicate ± standard deviations.

Table 4 shows the content of ustiloxin B in rice grain samples of the cultivars collected from different areas of China determined by icELISA and HPLC. For icELISA analysis, the samples collected from rice false smut disease regions were detected to have ustiloxin B, of which the content ranged from 1.03 to 97.90 µg/g. For HPLC analysis, only the grain samples of rice cultivars H 329, H 597 and Yanfeng 47 were detected to have ustiloxin B. Ustiloxin B was undetectable in the other samples (Table 4). The content of ustiloxin B in most of the rice grains agreed with the reported value [24]. The content of ustiloxin B in some rice grain samples was higher than the reported content, which might be due to the rice grains being mixed with rice false smut balls while harvest. The icELISA results were quite similar to those of HPLC (Table 4). A good correlation between the results of HPLC (*Y*) and icELISA (*X*) was observed with the linear regression equation of *Y* = 0.9226*X* + 2.0637 (*R*^2^ = 0.9792), which suggested that the developed icELISA was potentially an effective and accurate method for ustiloxin B analysis of rice grains, and the icELISA was more sensitive than the HPLC method.

By icELISA analysis, ustiloxin B content (170 to 710 μg/g) in rice false smut balls (Table 3) was seven- to 10,000-fold greater than that (1.03 to 97.90 μg/g) in rice grains (Table 4). In many rice cultivation areas, both people and livestock usually consume the rice grains contaminated with false smut balls and false smut pathogen, which requires attention to food and feed safety. Monitoring content of ustiloxins in rice grains and their products should be very important.

**Table 4:** Comparison of ustiloxin B content in rice grain samples (rice cultivars) detected by icELISA and HPLC.

Rice Cultivar (Collection Place and Time)	Ustiloxin B Content (μg/g)	
	icELISA	HPLC
H 329 (Donggang, Liaoning, China; November 2014)	23.89 ± 0.81 ^a^	28.28 ± 3.19 ^a^
H 597 (Donggang, Liaoning, China; November 2014)	44.14 ± 1.62	37.04 ± 0.50
Liaojing 212-14 (Donggang, Liaoning, China; November 2014)	1.03 ± 0.07	nd ^b^
Liaokai 79 (Donggang, Liaoning, China; November 2014)	1.84 ± 0.05	nd
Maisui 1 (Donggang, Liaoning, China; November 2014)	8.79 ± 0.04	nd
Shifangliaoyou (Donggang, Liaoning, China; November 2014)	4.65 ± 0.13	nd
Xiangjing (Donggang, Liaoning, China; November 2014)	2.28 ± 0.24	nd
Yanfeng 47 (Donggang, Liaoning, China; November 2014)	97.90 ± 2.64	93.96 ± 0.57
Yanjing 218 (Donggang, Liaoning, China; November 2014)	1.96 ± 0.15	nd
Tianyouhuazhan (Hanshou, Hunan, China; October 2013)	1.71 ± 0.06	nd
Lijiang (Shangzhuang, Beijing, China; October 2011)	nd	nd
Zhonghua 17 (Shangzhuang, Beijing, China; October 2013)	nd	nd

Note: ^a^ Data represent the4 means of triplicate ± standard deviations; ^b^ nd, not detected. The LOD/LOQ values from the ELISA used for ustiloxin B determination in rice grains were 30/125 ng/g; The LOD/LOQ values from the HPLC used for ustiloxin B determination in rice grains were 3.125/12.5 µg/g at signal-to-noise ratios (S/N) of about 3 and 10.

## 3. Experimental Section

### 3.1. Instruments

A Shimadzu Prominence LC-20A high-performance liquid chromatography system (Kyoto, Japan) consisted of two LC-20AT solvent delivery units, an SIL-20A autosampler, an SPD-M20A photodiode array detector, a CBM-20Alite system controller and a Synergi reversed-phase Hydro-C_18_ column (250 mm × 4.6 mm, 5 μm) (Phenomenex, Torrance, CA, USA).

Cell culture plates and 96-well polystyrene microtiter plates were purchased form Costar (Corning, NY, USA). The automatic plate washer (Wellwash 4 MK2, Thermo, Vantaa, Finland), direct heat CO_2_ incubator and microplate reader (Multiskan MK3) were purchased from Thermo (Vantaa, Finland). The electric heating constant temperature incubator was purchased from Tianjin Zhonghuan Experiment Electric Stove Co. Ltd. (Tianjin, China). The ultrasonic cleaner (KH-500E, Kunshan, Jiangsu, China) was purchased from Kunshan Hechuang Ultrasonic Apparatus Co. Ltd., Kunshan, Jiangsu, China.

### 3.2. Chemicals and Immunochemicals

Ustiloxin A (UA) and ustiloxin B (UB) were isolated and purified as described previously [18,33]. The reagents and chemicals, including cell freezing medium-dimethyl sulfoxide (DMSO) (serum-free), 8-azaguanine, hypoxanthine, aminopterin and thymidine (HAT), l-glutamine, penicillin, streptomycin, goat anti-mouse IgG conjugated with horseradish peroxidase (IgG-HRP), bovine serum albumin (BSA), ovalbumin (OVA), complete and incomplete Freund’s adjuvant, *N*,*N*-dimethylformamide (DMF) and *o*-phenylenediamine (OPD), were purchased from Sigma (St. Louis, MO, USA). Polyethylene glycol (PEG)-2000 was from Fluka (Buchs, Switzerland). Cell culture media (Dulbecco’s Modified Eagle’s Medium, DMEM) and fetal bovine serum (FBS) were obtained from Gibco BRL (PaisLey, Scotland). Methanol and trifluoroacetic acid (TFA) of chromatography gradient grade were purchased from Tianjin Tianhao Chemical Industry Co. Ltd. (Tianjin, China). All other reagents and solvents were of analytical grade.

Coating buffer (0.05 M carbonate buffer, pH 9.6), phosphate-buffered saline (PBS) (0.01 M phosphate buffer containing 0.9% NaCl, pH 7.5), PBS with 0.1% (*v/v*) Tween-20 (PBST), PBST containing 0.5% (*w/v*) gelatin (PBSTG), citrate-phosphate buffer (0.01 M citric acid and 0.03 M Na_2_HPO_4_, pH 5.5), substrate solution (4 μL of 30% H_2_O_2_ at *w/w* added to 10 mL of citrate-phosphate buffer containing 2 mg/mL OPD) and a stop solution (2 M H_2_SO_4_) used in the present work were the same as those previously published [32].

The HAT-sensitive Balb/c mouse myeloma cell line SP2/0 purchased from China Institute of Veterinary Drug Control (Beijing, China) was used in fusion experiments.

To ensure safety, all ustiloxins and contaminated samples (especially rice false smut balls) were placed in hermetic centrifuge tubes or plastic bags and stored in a refrigerator separately. The operator wore latex gloves and an anti-poison respirator during the whole process and washed their hands with a large amount of water after every experiment, as ustiloxins were water-soluble compounds.

### 3.3. Preparation of UB-Protein Conjugates

UB-OVA and UB-BSA conjugates were prepared via the glutaraldehyde method as an immunogen and coating antigen, the same as the previous reports [23,34]. Briefly, 2.3 mg of ustiloxin B dissolved in 1 mL of DMF were split into two equal volumes. Each was added to 1 mL of PBS containing OVA (7.54 mg) or BSA (11.35 mg) while stirring, followed by the addition of 6.8 µL of 5% glutaraldehyde solution into the mixture. The reaction mixtures were stirred overnight at 4 °C, dialyzed against PBS and stored at −20 °C.

The UV-VIS spectral data were used to confirm the structural characteristics of the final conjugates (UB-OVA and UB-BSA). The hapten densities (the number of hapten molecules per molecule of protein) of the conjugates were estimated directly by the extinction coefficient ε [35,36]. The absorbance of conjugation, protein and hapten was recorded at 280 nm and used for calculation of ε, respectively. ε was calculated as the absorbance of a 1 M solution of the substance. Hapten density = (ε_conjugation_ − ε_protein_)/ε_hapten_.

### 3.4. Immunization Protocol, Monoclonal Antibody Production and Purification

Seven female Balb/c mice (6 to 8 weeks of age) were immunized with UB-OVA conjugates mixed with Freund’s complete adjuvant or Freund’s incomplete adjuvant (*ca*. 100 µg per mouse; 50 µg was injected intraperitoneally, 50 µg was injected subcutaneously) at 2-week intervals. Three days after the fifth injections, the mice were retro-orbital bleeding, and sera were tested for anti-UB antibody titer and for UB recognition properties in icELISA. The mouse with the highest titer and best specificity was boosted intraperitoneally with 100 µg UB-OVA without adjuvant. Three days after the last injection, the spleen cells collected from it were fused with the SP2/0 cell line using PEG-2000 at a ratio of 10:1 of spleen to myeloma cells. Selective growth of the hybridoma cells was in complete medium (DMEM supplemented with 20% FBS (*v/v*), 0.2 M l-glutamine, 50,000 U/L penicillin, 50 mg/L streptomycin) with 1% (*v/v*) HAT for approximately two weeks at 37 °C in a CO_2_ incubator (5% CO_2_ in air). The supernatants were screened by icELISA. Positive hybridoma cells were cloned by limiting dilution, and clones were further selected by icELISA. The clone that had a high antibody titer and good sensitivity in the culture supernatant was expanded in mice for production of mAb in ascites. The mAbs were purified from ascites fluids by ammonium sulfate precipitation [32]. The immunoglobulin isotype was determined with a mouse antibody isotyping kit according to the instructions (Pierce, Rockford, IL, USA).

All of the experiments were approved by the Beijing Experimental Animal Management Office and performed in compliance with the regulation of Animals Welfare Act of the U.S. Department of Agriculture.

### 3.5. icELISA

#### 3.5.1. Establishment and Optimization of Conventional icELISA

The storage conditions of coating antigen, mAb, goat anti-mouse IgG-HRP, ustiloxin B and buffer solutions were the same as previously described [23]. Briefly, the coating antigen, purified mAb and goat anti-mouse IgG-HRP were dissolved in a mixed solution (50:50, *v/v*) of PBS and glycerol and stored at −20 °C. Ustiloxin B dissolved in sterile water was stored at 4 °C. The coating buffer, PBSTG and substrate solution were freshly prepared or stored at 4 °C for less than one week. The washing buffer (PBST) and stop solution (2 M H_2_SO_4_) were preserved at ambient temperature.

The procedure of icELISA was the same as previously described with minor modifications [23]. A 96-well microplate was coated with 100 µL of coating antigen (UB-BSA) solution (0.5 µg/mL) per well in coating buffer at 37 °C for 3 h and then washed with PBST four times on an automatic plate washer. Fifty microliters of various concentrations of the standard or analyte solution diluted in PBSTG were pipetted into each well, followed by the addition of 50 μL of sera, supernatant or purified mAb solution diluted in PBSTG. After being incubated at 37 °C for 30 min, the plate was washed with PBST four times to remove the unbound antibodies. An aliquot of 100 μL goat anti-mouse IgG-peroxidase conjugate diluted in PBSTG (1 μg/mL) was added to each well. The plate was incubated at 37 °C for 30 min and then washed with PBST again. One hundred microliters of substrate solution were added into each well. Then, the reaction was terminated by adding 50 μL of the stop solution (2 M H_2_SO_4_) per well. Absorbance was read at 492 nm on a Multiskan MK3 microplate reader [32].

To optimize the conventional icELISA, various dilutions of the coating antigen UB-BSA (0.06 to 2.00 µg/mL) and mAb 1B5A10 (0.13 to 2.00 µg/mL) were screened by checkerboard titration [23]. The calibration curve data were imported into OriginPro 8.0 (OriginLab) and fit to a sigmoidal logistic equation.

#### 3.5.2. Cross-Reactivity Study

The mAb specificity was estimated by cross-reactivity (CR) with ustiloxin A via icELISA. As the structure of ustiloxin A is very similar to that of ustiloxin B (Figure 1), ustiloxin A was selected for CR estimation of the mAb IB5A10. CR of ustiloxin A was calculated according to the formula: CR (%) = [IC_50_ (ustiloxin B)/IC_50_ (ustiloxin A)] × 100.

#### 3.5.3. Sample Extraction and Recovery Studies

The rice false smut ball sample collected from Hanshou and the rice grain sample obtained from the disease-free region (Beijing, China) were used for ustiloxin B recovery studies. The rice grain sample was the uncontaminated sample, which was determined by HPLC. Collected samples (50 to 120 g) were ground and mixed. Then, 0.1 g (rice false smut ball) and 1 g (rice grain) of the mixed samples were weighed for analysis. The samples were extracted according to the procedure previously described [18].

The ustiloxin B was dissolved in ultrapure water as the standard solution of 2 mg/mL for calibration and spiking experiments. A series of concentrations of ustiloxin B was diluted with PBSTG (0, 3.125, 6.25, 12.5, 25, 50, 100 and 200 ng/mL) and ultrapure water (0, 3.125, 6.25, 12.5, 25, 50, 100 and 200 μg/mL) to protract the calibration curve of icELISA and HPLC, respectively.

For rice false smut ball samples, each 0.1 g of powdered sample spiked with ustiloxin B at contents of 0, 0.2, 0.4, 0.8, 1.6 and 3.2 mg/g, respectively, was extracted with ultrapure water three times (3 × 1.5 mL, 30 min each time) in an ultrasonic bath at room temperature, followed by centrifugation at 6710× *g* for 15 min. The supernatant extracts were combined and adjusted to 5.0 mL with ultrapure water. The extract was divided into two equal aliquots. One aliquot was diluted 800-fold with PBSTG, followed by analysis with icELISA in triplicate. The spiked concentrations of ustiloxin B in the diluted extracted solutions were at 0, 5, 10, 20, 40 and 80 ng/mL. The other aliquot was filtered through a filter (pore size, 0.22 μm) and analyzed on a Shimadzu LC-20A high-performance liquid chromatograph system that consisted of two LC-20AT solvent delivery units, an SIL-20A autosampler, an SPD-M20A photodiode array detector, a CBM-20Alite system controller and a Synergi reversed-phase Hydro-C_18_ column (250 mm × 4.6 mm, 5 μm). The injection volume was 30 μL. The mobile phase, composed of methanol:water (15:85, *v/v*) containing 0.02% TFA (*v/v*), was set at a flow rate of 1.0 mL/min, in isocratic elution mode at a temperature of 40 °C. The detection wavelength was at 220 nm. The total analysis time was 25 min [18]. The spiked concentrations of ustiloxin B in the combined extracts were 0, 4, 8, 16, 32 and 64 µg/mL.

For rice grain samples, each 1.0 g of the powered sample spiked with ustiloxin B at concentrations of 0, 100, 250, 500, 1000, 2000, 4000 and 5000 ng/g was extracted with ultrapure water three times (3 × 6.0 mL, 30 min each time) in an ultrasonic bath at room temperature. The extracts were combined and then concentrated by a vacuum freeze dryer to dryness, and the residue was dissolved in 1 mL of ultrapure water in a test tube [23]. The extracted solution was divided into two aliquots. One aliquot (200 µL) was diluted 50-fold with PBSTG, followed by analysis with icELISA in triplicate. The spiked concentrations of ustiloxin B in the diluted extracted solutions were at 0, 2, 5, 10, 20, 40, 80 and 100 ng/mL. The other aliquot (800 µL) of concentrated solution was then filtered through a filter (pore size, 0.22 μm) and analyzed by HPLC. The conditions of HPLC were exactly the same as described above (for rice false smut ball samples). The spiked concentrations of ustiloxin B in the extracted solutions were 0, 100, 250, 500, 1000, 2000, 4000 and 5000 ng/mL.

### 3.6. icELISA and HPLC Analysis of Ustiloxin B in Rice False Smut Ball Samples and Rice Grain Samples

The rice false smut ball samples were collected from different areas of China or at different periods of rice false smut disease, and the rice grain samples were collected from different rice cultivars in different areas of China. The samples were extracted with ultrapure water according to the procedure described above (Section 3.5.3.) with minor modifications.

**Figure 3:**
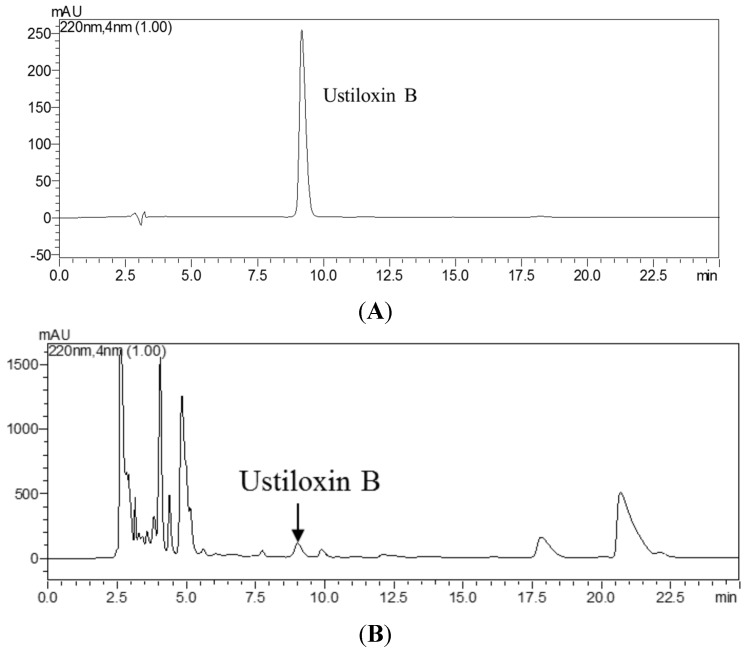

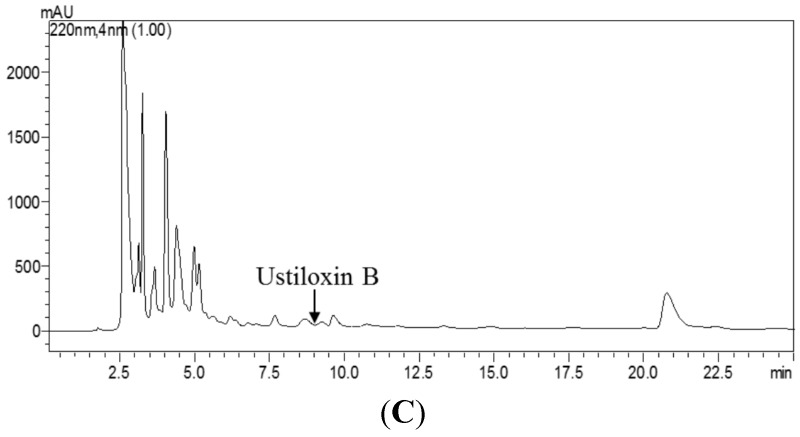
HPLC analysis of ustiloxin B in rice false smut balls. (**A**) HPLC profile of the reference ustiloxin B; (**B**) HPLC profile of the water extract of rice false smut balls from Linyi, Shandong (0.52 mg/g); (**C**) HPLC profile of the water extract of rice grains from Donggang, Liaoning of China (<10 µg/g).

For rice false smut ball samples, 0.1 g of powdered sample was extracted with ultrapure water three times (3 × 1.5 mL, 30 min each time) in an ultrasonic bath at room temperature, followed by centrifugation at 6710× *g* for 15 min. The supernatant extracts were combined and concentrated by a vacuum freeze dryer to dryness, and the residue was dissolved in 1 mL of ultrapure water in a test tube. The extracted solution was divided into two aliquots. One aliquot (100 µL) was diluted 2000-fold with PBSTG, followed by analysis with icELISA in triplicate. The other aliquot (900 µL) of concentrated solution was then filtered through a filter (pore size, 0.22 μm) and analyzed by HPLC. Figure 3 shows the HPLC analysis of ustiloxin B in rice false smut balls and rice grains. The conditions of HPLC were the same as described above (Section 3.5.3.). The retention time of ustiloxin B was 9.17 min. The HPLC calibration curves of ustiloxin B showed a good linearity, *Y* = 41,981.5058*X* − 19,568.6954, *R*^2^ = 1.0000, where *Y* is the peak area of analyte and *X* is the concentration (μg/mL) of analyte.

For rice grain samples, the extraction was the same as the procedure for rice grain samples described above (Section 3.5.3.) without the addition of ustiloxin B.

## 4. Conclusions

In this study, ustiloxin B, an antimitotic cyclopeptide mycotoxin, as well as the second most active ustiloxin in the rice false smut balls, was used to produce monoclonal antibodies to develop an icELISA for ustiloxin B analysis. Ustiloxin B was conjugated with OVA and BSA via the glutaraldehyde method to prepare an immunogen and coating antigen, respectively. The mAb 1B5A10 recognized ustiloxin A with a cross-reactivity of 13.9%. A more specific monoclonal antibody against ustiloxin B would be required if we want to develop an ELISA more specific for ustiloxin B. The content of ustiloxin B in the samples including rice false smut balls and rice grains determined by icELISA agreed well with that by HPLC. The recovery study, as well as the comparison study of icELISA and HPLC demonstrated that the developed icELISA was a reliable and accurate analysis method for ustiloxin B. Furthermore, the established icELISA was more sensitive, lower cost, less time consuming and higher throughout in comparison to HPLC, which indicated that icELISA is suitable for routine screening and monitoring the content of ustiloxin B in rice false smut balls, as well as rice food and feed samples.
